# Placenta Prævia Treated by Digital Compression

**Published:** 1872-01

**Authors:** 


Placenta Prævia Treated by Digital Compression—Dr. R. H. Plummer, in Pacific Medical and Surgical Journal, details a case in which he was able to save the life of a woman, during delivery of a placenta attached at the os, by digital compression of the arteries. In the case that he describes, and those of like nature, we should regard it as a valuable adjunct to the treatment of this terrible complication.

